# Laser-Patternable Graphene Field Emitters for Plasma Displays

**DOI:** 10.3390/nano9101493

**Published:** 2019-10-19

**Authors:** Kamatchi Jothiramalingam Sankaran, Santosh Kumar Bikkarolla, Derese Desta, Susanta Sinha Roy, Hans-Gerd Boyen, I-Nan Lin, James McLaughlin, Ken Haenen

**Affiliations:** 1 Institute for Materials Research (IMO), Hasselt University, 3590 Diepenbeek, Belgium; derese.desta@uhasselt.be (D.D.); hansgerd.boyen@uhasselt.be (H.-G.B.); 2 IMOMEC, IMEC vzw, 3590 Diepenbeek, Belgium; 3 School of Engineering, Engineering Research Institute, University of Ulster, Newtownabbey BT37 0QB, UK; bikkarolla@gmail.com (S.K.B.); jad.mclaughlin@ulster.ac.uk (J.M.); 4 Department of Physics, School of Natural Sciences, Shiv Nadar University, Uttar Pradesh 201314, India; susanta.roy@snu.edu.in; 5 Department of Physics, Tamkang University, Tamsui 251, Taiwan, China; inanlin@mail.tku.edu.tw

**Keywords:** laser-induced graphene, polyimide, field electron emission, plasma illumination

## Abstract

This paper presents a plasma display device (PDD) based on laser-induced graphene nanoribbons (LIGNs), which were directly fabricated on polyimide sheets. Superior field electron emission (FEE) characteristics, viz. a low turn-on field of 0.44 V/μm and a large field enhancement factor of 4578, were achieved for the LIGNs. Utilizing LIGNs as a cathode in a PDD showed excellent plasma illumination characteristics with a prolonged plasma lifetime stability. Moreover, the LIGN cathodes were directly laser-patternable. Such superior plasma illumination performance of LIGN-based PDDs has the potential to make a significant impact on display technology.

## 1. Introduction

Displays are an essential interface in machine-based communication. There have been major developments in display technology, with the potential to enable television, handheld computers, and mobile phones to be more functional and user-friendly [1,2,3,4]. In this regard, plasma display devices (PDDs) are very attractive for display technology. The advantages of plasma display are sharper image, wider screen option, better contrast ratios, high-definition quality, less visible motion blurs, superior uniformity, and wider viewing angle than cathode ray display and liquid crystal displays [3,4,5]. However, their relatively high operating voltage and poor plasma stability have limited their widespread use [6,7]. To mitigate these issues, several studies have been conducted to find a suitable cathode material, which possesses a high proficiency in producing secondary electrons through plasma ion bombardment for a longer duration. Theoretical works from Venkatraman et al. [7,8] advise that a material with outstanding field electron emission (FEE) properties is appropriate as a cathode in improving the characteristics of a PDD. 

Graphene, two-dimensional hexagonally arrayed carbon atoms, is considered a viable electron emitter for FEE applications as the sharp edges of individual graphene sheets are high-density sources of individual field emission sites [9]. However, a requirement for the graphene utilized in field electron emission devices is that the material should be vertically aligned/protrude from the polymer substrate, providing more individual field emission sites, as flat graphene sheets lack sharp edges and require a high voltage to turn on the FEE process [10]. Several methods for synthesizing graphene nanostructures on polymer substrates, such as spin-casting, electrophoresis, self-assembly, thermal welding, and filtering, have been developed and have employed the obtained nanostructures as efficient field emitters [11,12,13,14,15,16]. A cost-effective process of synthesizing graphene nanostructures on polymers using cheap precursors is needed for the industrial production of display devices.

In this work, such a cost-effective method is reported for the synthesis of graphene nanoribbons using the laser induction process on commercially accessible polyimide sheets. The obtained graphene nanoribbons were successively utilized as a cathode for fabricating field emission and plasma display devices. The detailed morphological and structural features of the developed material were investigated. Superior plasma illumination properties with low breakdown field and prolonged plasma stability were achieved for PDDs utilizing graphene nanoribbons as cathodes. The better plasma illumination properties for the PDDs were correlated with the FEE properties of the graphene nanoribbons.

## 2. Materials and Methods 

**Figure 1:**
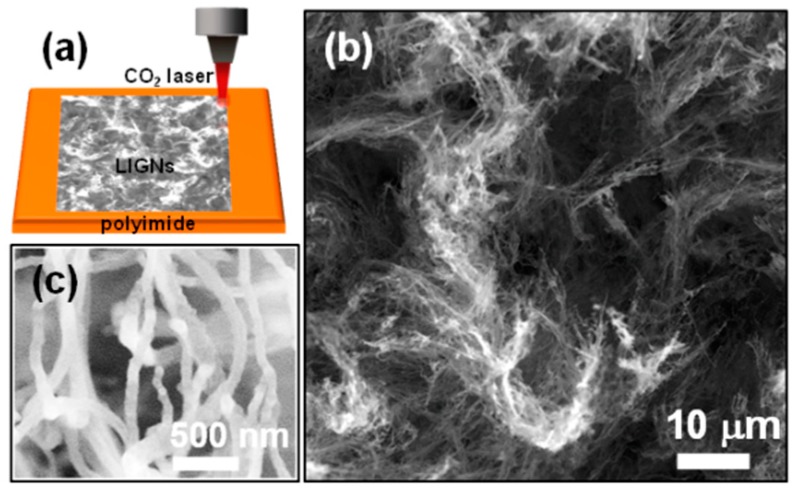
(**a**) Schematic illustration showing the fabrication process of the laser-induced graphene nanoribbons (LIGNs), (**b**) plan-view field emission scanning electron microscopy (FESEM) micrograph of the LIGNs, (**c**) high-resolution FESEM micrograph of the LIGNs.

Laser induction on polyimide sheets, schematically shown in Figure 1a, was performed using Universal Laser Systems VLS2.30 equipped with a wavelength of 10.6 µm pulsed CO_2_ laser system (25 W). Polyimide sheets with a thickness of 0.125 mm (Cat. No: IM301450) were purchased from Goodfellow, Huntingdon, England. A scan rate of 20 cm/s, a laser duty cycle of 30%, and an image density of 1000 ppi were used to obtain a black layer of laser-induced graphene nanoribbons (LIGNs) on the polyimide sheets [17,18]. The grown LIGNs were characterized using field emission scanning electron microscopy (FESEM; SU5000 (Hitachi High-Technologies Corporation, Tokyo, Japan)), transmission electron microscopy (TEM; Jeol 2011 at 200 kV accelerating voltage (Jeol Taiwan Semiconductors Limited, Hsinchu, Taiwan)), Raman spectroscopy (Renishaw confocal microscope; λ = 532 nm, Paris, France), X-ray photoelectron (PHI 6000; Al Kα radiation with an energy of 1486.6 eV and an energy resolution of 0.47 eV, Physical Electronics, Chanhassen, MN, USA) spectroscopy, and X-ray diffraction (XRD; Bruker D8-discover diffractometer fitted with global mirror (Cu Kα radiation source, λ = 1.540 Å); Coventry, UK). The TEM samples were prepared by removing the LIGN films from the polyimide sheets, followed by ultrasonication in absolute ethanol and coating the TEM grids with a few microliters of the solution.

To estimate the ability of the LIGNs as field emitters, the distance between a Mo rod with a diameter of 3 mm (anode) and the LIGNs (cathode; 1 cm × 1 cm) was set to 640 μm, and the current–voltage characteristics were measured using a Keithley 6517B electrometer (Keithley Instruments, Inc., OH, USA) inside a vacuum chamber around a pressure of 1.5 × 10^−8^ Torr. To examine the plasma illumination (PI) characteristics of a plasma display device, a cylindrical-type plasma device was fabricated. The cathode was the LIGNs, and an indium tin oxide (ITO)-coated glass was used as an anode. The separation between the cathode and anode was fixed by a 1.0 mm thick polytetrafluoroethylene (PTFE) spacer. A cylindrical cavity with a diameter of 8.0 mm was formed in the PTFE spacer. The whole device was placed in a vacuum chamber, and a pressure of 0.01 mTorr was maintained. Argon gas at a flow rate of 10 sccm was passed into the chamber during the measurements. A DC pulsed voltage (HPP-20KA01KAT B; continuous 325–1000 VDC; Delta Electronics Inc., Taiwan, China) was used to ignite the plasma under 10 Torr, and a Keithley 2410 electrometer was employed to measure the plasma current density (*J*_PI_)–applied field (*E*) characteristics.

## 3. Results and Discussion

**Figure 2:**
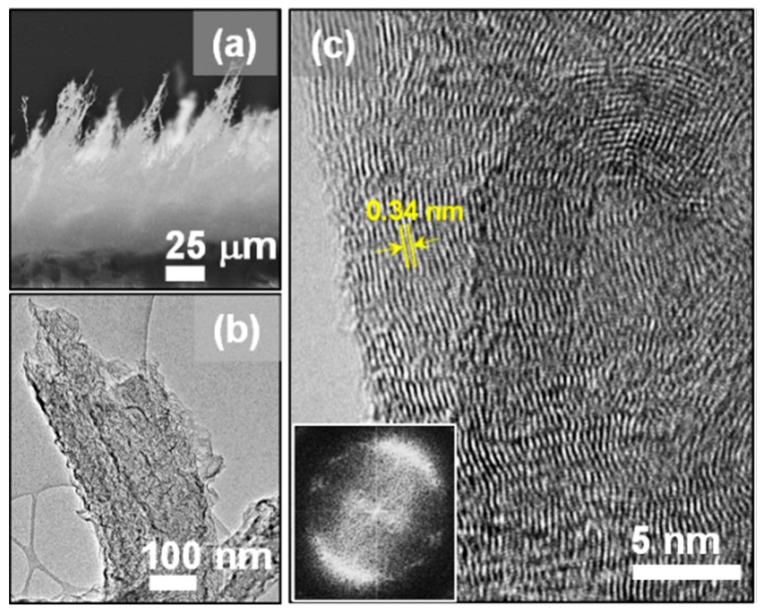
(**a**) Cross-sectional view FESEM micrograph of laser-induced graphene nanoribbons (LIGNs). (**b**) TEM micrograph and (**c**) high-resolution TEM (HRTEM) structural image of the LIGNs. The inset in (**c**) shows the Fourier transformed image corresponding to the whole structural image in (**c**).

Imaging of the LIGN surface using FESEM (Figure 1b) displayed the three-dimensional nature of foam-like graphene. The high-magnified SEM micrograph (Figure 1c) revealed that the graphene was composed of interconnected nanoribbons. Additional FESEM micrographs (given in Figure S1 of the Supplementary Materials) indicated a homogenous morphology of the nanoribbons in the graphene film, and the average width of the graphene nanoribbons was ~100 nm. The thickness of the LIGNs, estimated from cross-sectional SEM image (Figure 2a), was 120 μm. Moreover, sharp edges of nanoribbons were observed, which were spiked-out from the surface of the substrate. The microstructure of the LIGNs was revealed by TEM. The TEM micrographs (Figure 2b) disclosed that the nanoribbons with the width of ~100–250 nm contained nanoscale ripples and wrinkles. From the high-resolution TEM micrograph (Figure 2c), it could be seen that the LIGNs displayed a few-layered graphene structure with a d-spacing of 0.34 nm, representing (002) planes, with numerous graphene edges. Moreover, the Fourier transformed diffractogram corresponding to the whole high-resolution TEM micrograph (displayed as an inset in Figure 2c) revealed a donut-shaped strong diffuse ring, indicating the presence of graphene phase in the material. The nanoscale ripples and wrinkles observed in TEM were formed due to the thermal expansion that happened via laser irradiation. The formation of graphene by the laser was like a photothermal process [18] as a long wavelength and long pulse laser was used in this study. 

**Figure 3:**
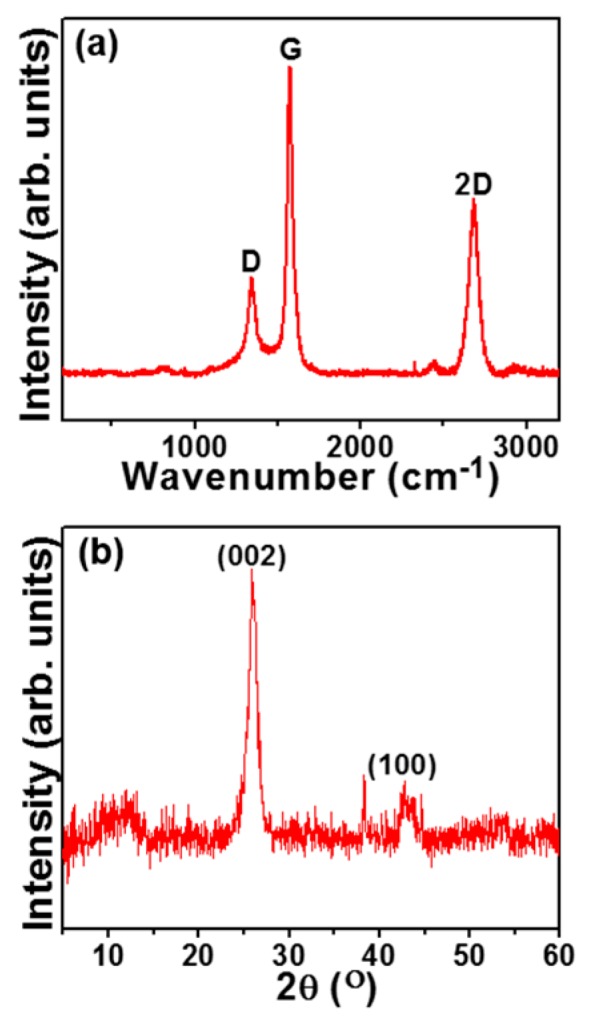
(**a**) Raman (λ = 532 nm) spectrum and (**b**) X-ray diffraction spectrum of the LIGNs.

The structural quality of the LIGNs was evaluated by Raman spectroscopy. The Raman spectrum of the LIGN, shown in Figure 3a, was composed of three major peaks: the typical D, G, and 2D [17,18]. The presence of a small D-peak at 1342.7 cm^−1^ was related to the sp^3^ centers in the LIGN due to the structural edge defects. The 2D peak at 2680 cm^−1^ was fitted with only one Lorentz peak of width 61.3 cm^−1^, similar to monolayer graphene [19]. The I_D_/I_G_ ratio of 0.34 specified a high degree of sp^2^ network in the LIGNs, and the average I_2D_/I_G_ ratio of 0.53 indicated the presence of multilayered graphene, which is consistent with the HRTEM (cf. Figure 1b,c). Moreover, the XPS spectrum of the LIGNs (Figure S2a of the Supplementary Materials) showed a high carbon peak of 96.3 at.% and a small oxygen peak of 3.7 at.%. The XRD spectrum (Figure 3b) showed a peak at 25.96°, representing an interlayer distance of 0.34 nm of (002) planes in the LIGN, indicating a high degree of crystallinity [17,18]. The slight increase in the interlayer spacing represented the existence of defects in the graphene sheets. A peak at 43.1° corresponded to (100) planes, which was attributed to the in-plane structure of the graphene sheet. Taken together, the characterization studies confirmed that the fabricated material was indeed LIGNs.

**Figure 4:**
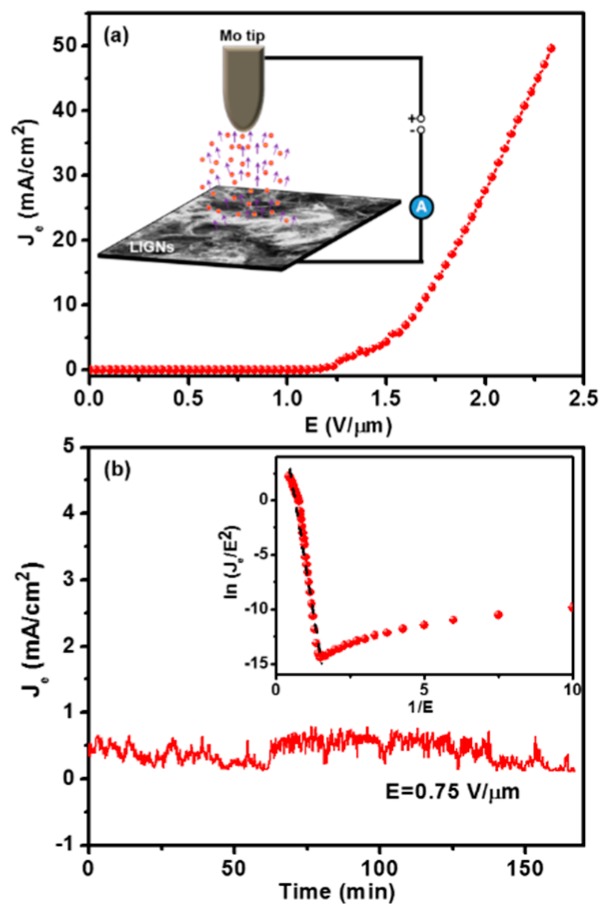
(**a**) Field electron emission properties (current density–applied field (*J*_e_*–E*) curves) measured in a high vacuum environment for LIGNs, with the inset showing the schematic of the FEE measurement. (**b**) Lifetime stability measurements (*J*_e_–time curves) for LIGNs, with the inset showing the Fowler–Nordheim (F–N) plots corresponding to the *J–E *curves shown in (**a**).

Figure 4a shows the FEE current density (*J*_e_) versus electric field (*E*) characteristics for the LIGNs, with the inset of Figure 4a presenting a schematic of the FEE measurement set-up of the LIGNs. The turn-on field (*E*_0_) was described as the electric field essential to attain a current density of 10 μA/cm^2^. The LIGNs exhibited a low *E*_0_ value of 0.44 V/μm, with a high *J*_e_ value of 49.7 mA/cm^2^ at an applied field of 2.33 V/μm. Fowler–Nordheim (F–N) theory was used to explain the FEE characteristics of the materials [20].

(1) $$J_{e\;}=\left(\frac{A\beta^{2}E^{2}}{\varphi }\right)\exp\left(-\frac{B\times \varphi^{\frac{3}{2}}}{\beta \times E}\right)$$

where *A* = 1.54 × 10^−6^ A eV/V^2^ and *B* = 6.83 × 10^9^ eV^−3/2^ V/m, *φ* is the work function, and *β* is the field-enhancement factor of the emitting materials. The slope (*m*) of the F–N plot (inset of Figure 3b) provides the corresponding *β* value using the formula *β* = [−6.8 × 10^3^ × *φ*^3/2^]/*m*. In order to calculate the *β* value of the LIGNs, an average *φ* value of 3.614 eV was determined using Kelvin probe force microscopy (KPFM) (Figure S3 of the Supplementary Materials), which was lower than the reported *φ* value of graphene nanostructures [21,22,23,24,25]. Using this *φ* value, a *β* value of 4578 was calculated for the LIGNs. The FEE properties of the LIGNs were excellent and comparable with those of other reported field emitters (Table S1 of the Supplementary Materials). Generally, LIGNs possess prominent, vertically aligned sharp-edged graphene materials (cf. Figure 2a), resulting in a high aspect ratio [17,18,26,27,28,29,30,31,32,33,34,35,36,37] and therefore excellent FEE performance. The *J*_e_ versus time curve, measured at an applied field of 0.75 V/μm (Figure 4b), showed that the FEE current density was very stable for a period of 160 min. This confirms that LIGNs have a high FEE current stability, which is a beneficial characteristic for device applications.

**Figure 5:**
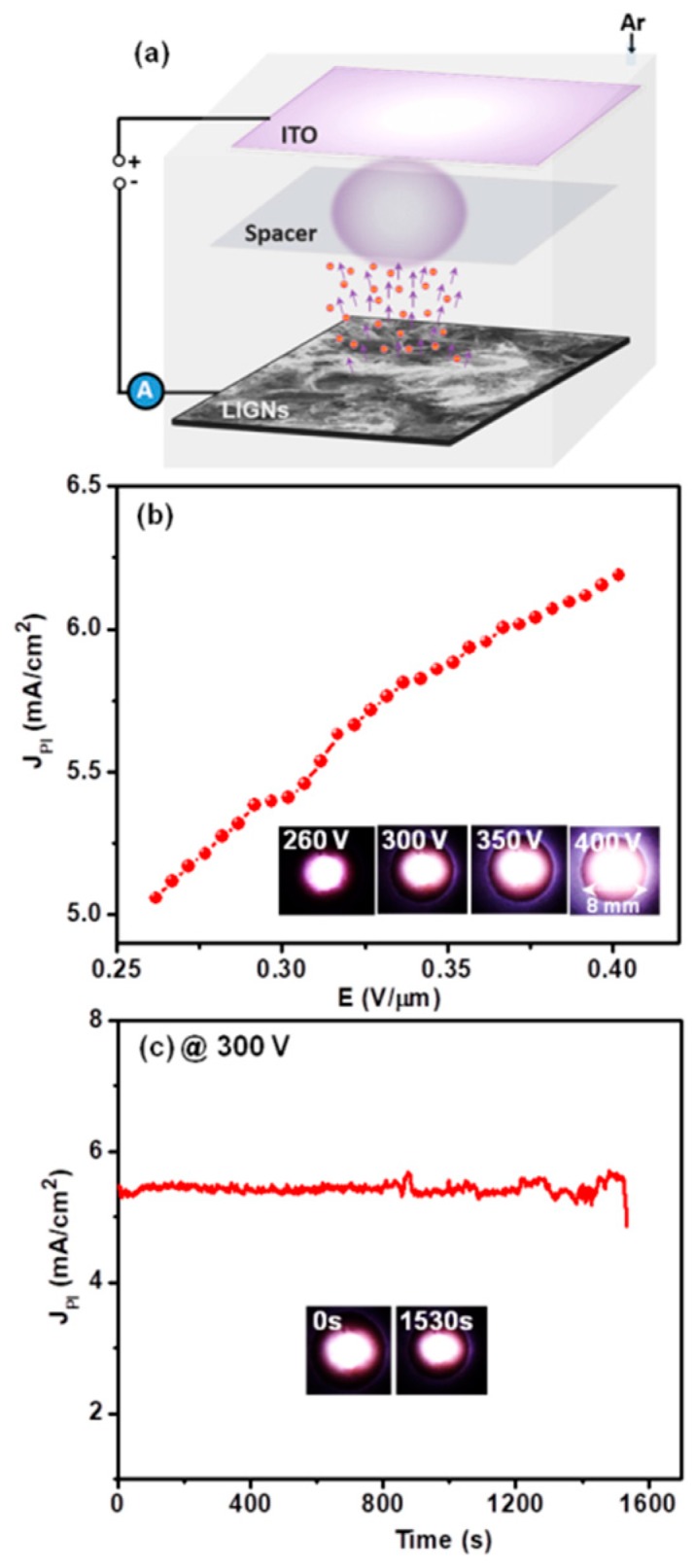
(**a**) Schematic of the plasma display device. (**b**) The plasma current density (*J*_PI_) versus applied field (*E*) of a plasma display device. Inset of (**b**) shows the photographs of plasma illumination characteristics of the plasma display device at varying voltages. (**c**) The plasma illumination stability of the LIGNs at an applied voltage of 300 V, displaying the plasma illumination intensity at 0 s and 1530 s after ignition of plasma (inset of (**c**)).

The superior FEE characteristics of the LIGNs play a beneficial role in plasma displays. Figure 5a displays a schematic illustration of the PI measurements, and Figure S4 of the Supplementary Materials shows a photograph of the homemade PI instrument. The PI images were obtained for different applied voltages through the anode by a USB microscope, and the PI behavior of the plasma display device was characterized. The series of PI images, shown in the inset of Figure 5b, revealed that the brightness of the plasma increased with the increase in applied voltage. The LIGN-based plasma display device needed a low breakdown voltage of 260 V (breakdown field (*E*_bk_) of 0.26 V/μm) to trigger the plasma. A *J*_PI_ value of 6.2 mA/cm^2^ was also achieved for the LIGN-based plasma display device at an applied field of 0.40 V/μm. Furthermore, the stability of the LIGNs as a cathode in a plasma display device was evaluated by applying a contact voltage of 300 V (a *J*_PI_ value of 5.3 mA/cm^2^). Interestingly, the LIGNs showed a stable plasma current over 25 min (1530 s) (inset of Figure 5c), and the intensity of the plasma also remained stable after 25 min (plasma images in the inset of Figure 5c), demonstrating the high robustness of the LIGNs.

**Figure 6:**
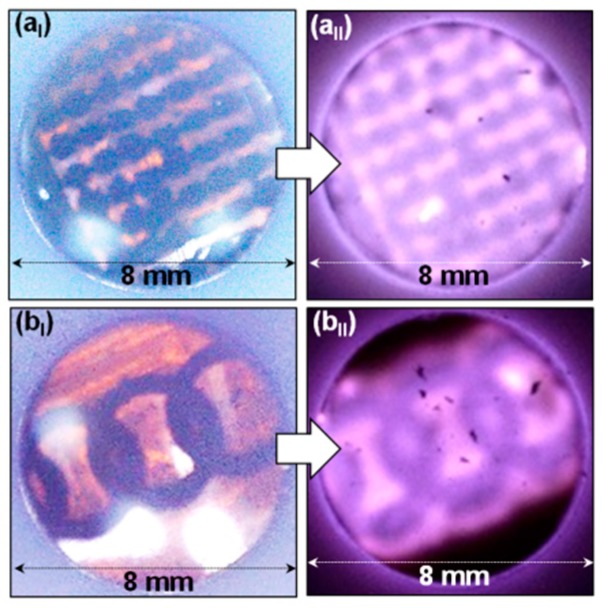
Patterned lines (**a_I_**) and circles (**b_I_**) of the LIGNs on polyimide substrates and (**a_II_,b_II_**) the corresponding plasma images of the LIGN-based plasma display devices at an applied voltage of 350 V.

Furthermore, LIGN-based patterned lines and circles were designed on polyimide substrates (shown in Figure 6a_I_,b_I_, respectively) and utilized as cathodes in plasma display devices. The plasma images at an applied voltage of 350 V (a *J*_PI_ value of 5.88 mA/cm^2^) (shown in Figure 6a_II_,b_II_, respectively) revealed the uniform lighting pattern of the LIGN-based plasma display devices. These results illustrate the overwhelming advantage of the LIGN materials, viz. they are directly laser-patternable, a characteristic that has not been achievable when using other kinds of materials as cathodes in plasma devices. Consequently, the benefit of the superior FEE properties as emitters reveals high robustness and high PI intensity for these materials, rendering LIGNs marvelous potential for application as plasma display device cathodes.

## 4. Conclusions

To summarize, we have reported on a high-performance plasma display device with a LIGN cathode architecture via a cost-effective method of direct laser scribing of polyimide sheets. The Raman, XRD, and TEM studies confirmed that the LIGNs contained multilayered graphene layers. The excellent FEE characteristics demonstrated the potential to use LIGNs as a cathode for plasma display devices. The LIGN-based plasma display device showed a low breakdown field of 0.26 V/μm, a high plasma current density of 6.2 mA/cm^2^, and a prolonged plasma lifetime stability of 25 min at an operating current density of 5.3 mA/cm^2^ with a stable plasma intensity. Moreover, the LIGN cathodes were found to be directly laser-patternable. Considering the simple and direct way of creating laser-fabricated LIGN-based plasma display devices, this work sets the basis for high-brightness display devices.

## Supplementary Materials

The following are available online at https://www.mdpi.com/2079-4991/9/10/1493/s1, Figure S1: FESEM micrographs of the LIGNs. Figure S2: (**a**) The X-ray photoelectron spectroscopic (PHI 6000; Al Kα radiation with an energy of 1486.6 eV and an energy resolution of 0.47 eV) survey spectrum shows a dominant carbon peak and a small oxygen peak of 96.3 at.% and 3.7 at.%, respectively, along with (**b**) the C1s and (**c**) O1s XPS spectra of the LIGNs. Figure S3: (**a**) Surface topography and (**b**) work function map of the LIGNs. Figure S4: Photograph of the homemade PI instrument.
