# Supplementary material for: Laser-Patternable Graphene Field Emitters for Plasma Displays
Source: Nanomaterials (Basel). 2019 Oct 19;9(10):1493. doi: 10.3390/nano9101493 (PMC6835302; doi:10.3390/nano9101493)
Supplement: Supplementary file 1 [file nanomaterials-09-01493-s001.pdf]

Supplementary Information

Laser patternable graphene field emitters for plasma displays

Kamatchi Jothiramalingam Sankaran[1,2,a,*], Santosh Kumar Bikkarolla[3,a], Derese Desta[1,2], Susanta Sinha Roy[4], Hans-Gerd Boyen[1,2], I-Nan Lin[5], James McLaughlin[3], Ken Haenen[1,2,*]

[1] Institute for Materials Research (IMO), Hasselt University, Diepenbeek, Belgium; derese.desta@uhasselt.be (D.D.); hansgerd.boyen@uhasselt.be (H.G.B.)
[2] IMOMEC, IMEC vzw, Diepenbeek, Belgium.
[3] School of Engineering, Engineering Research Institute, University of Ulster, Newtownabbey BT37 0QB, UK; bikkarolla@gmail.com (S.K.B.); jad.mclaughlin@ulster.ac.uk (J.M.L.)
[4] Department of Physics, School of Natural Sciences, Shiv Nadar University, Uttar Pradesh 201314, India; susanta.roy@snu.edu.in (S.S.R.)
[5] Department of Physics, Tamkang University, Tamsui 251, Taiwan, Republic of China; inanlin@mail.tku.edu.tw (I.N.L.)
[a] K.J.S. and S.K.B. contributed equally to this work.
[*] Correspondence: sankaran.kamatchi@uhasselt.be, ken.haenen@uhasselt.be; Tel: +32-11-268-826

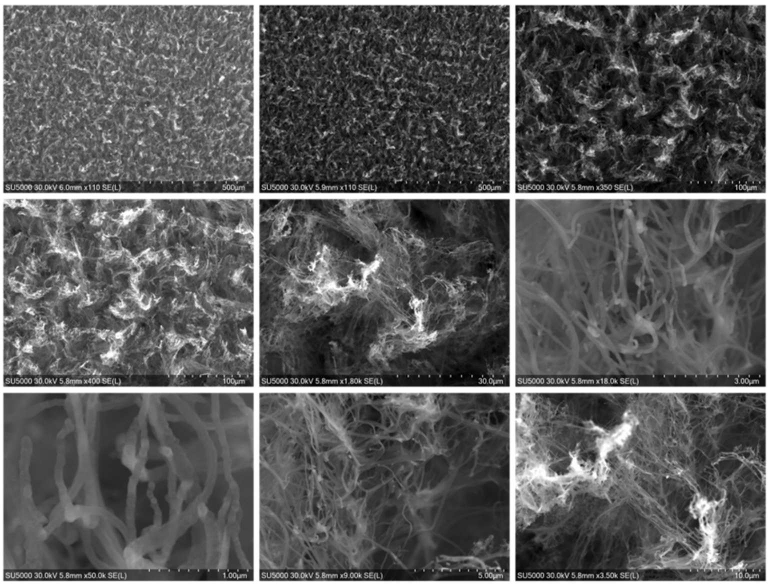

Figure S1. FESEM micrographs of the LIGNs, indicating the homogenous morphology of the nanoribbons in graphene film.

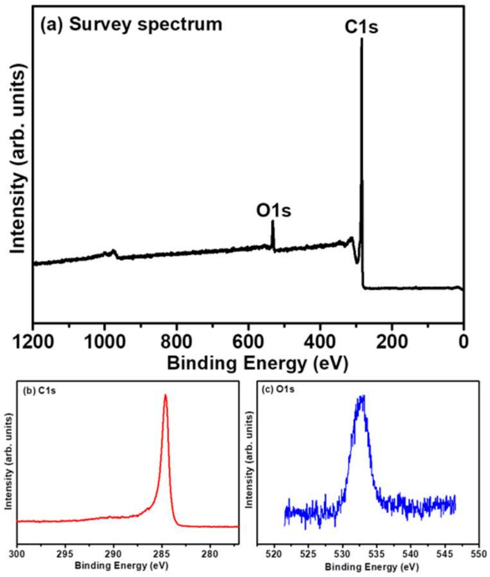

Figure S2. (a) The X-ray photoelectron spectroscopic (PHI 6000; Al Kα radiation with an energy of 1486.6 eV and an energy resolution of 0.47 eV) survey spectrum of the LIGNs shows dominant carbon and a small oxygen peaks of 96.3 at.% and 3.7 at.%, respectively. (b) C1s and O1s spectra of the LIGNs.

Kelvin Probe Force Microscopy (KPFM) measurements were performed to find the work function of LIGNs using a Park NX-10 Atomic force microscope equipped with PPP-EFM probes (Nanosensors). In KPFM the cantilever is driven in non-contact regime at a frequency slightly larger than the fundamental resonant frequency and an oscillation amplitude set point of 18 nm. An AC bias of 2 V amplitude at a frequency of 17 kHz was applied to the cantilever to probe electrostatic forces. The contact potential difference (V_{CPD}) between the conducting AFM tip and the sample is,

$$V_{CPD} = \frac{\varphi_{Tip} - \varphi_{Sample}}{-e} \quad (1)$$

where e is the electronic charge, φ_{Tip} and φ_{Sample} are the work functions of the AFM tip and the LIGNs film, respectively.

After determining the work function of the AFM tip using a freshly exfoliated highly oriented pyrolytic graphite (HOPG) film of a known work function (4.65 eV) [S1], the work function of the LIGNs was calculated using the relation,

$$\varphi_{LIGNs} = \varphi_{Tip} + eV_{CPD} \quad (2)$$

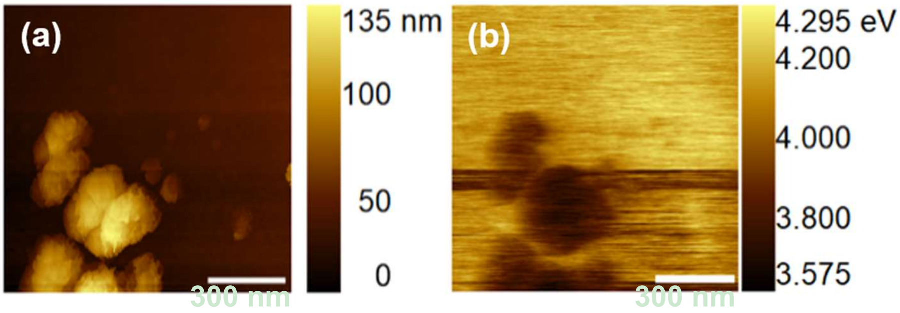

Figure S3. (a) Surface topography and (b) work function map of the LIGNs.

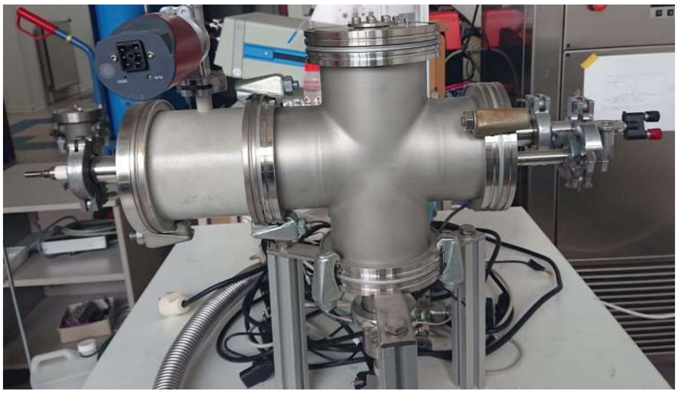

Figure S4. Photograph of the homemade PI instrument.

Table S1. Comparison between the field electron emission characteristics of LIGNs and other field emitters.

Materials	Turn-on field (V/μm)	Field enhancement factor	Lifetime (min)
VS_2 nanosheet [S2]	1.4	5588	240
Metallic nanowire-graphene hybrids [S3]	---	3533	0.8
BaO nanostructures [S4]	1.24	2458	---
Carbon-doped ZnO film [S5]	18.0	474	61
Diamond-like carbon films [S6]	40.0	---	---
SiC nanowires [S7]	0.95	4670	360
Multi-walled carbon nanotubes [S8]	4.1	1546	---
Single-walled carbon nanotubes [S9]	2.8	3066	
Diamond pyramidal microtips [S10]	1.8	4580	210
LIGNs [Present study]	0.44	4578	160

References

[S1] Sommerhalter, C.; Matthes, T.W.; Glatzel, T.; Jager-Waldau, A.; LuxSteiner, M.C. High-sensitivity quantitative Kelvin probe microscopy by noncontact ultra-high-vacuum atomic force microscopy. *Appl. Phys. Lett.* 1999, *75*, 286-288. DOI.org/10.1063/1/124357.

[S2] Song, C.; Yu, K.; Yin, H.; Fu, H.; Zhang, Z.; Zhang, N.; Zhu, Z. Highly efficient field emission properties of a novel layered VS_2/ZnO nanocomposite and flexible VS2 nanosheet. *J. Mater. Chem. C.* **2014**, *2*, 4196–4202 DOI. 10.1039/C4TC00025K.

[S3] Arif, M.; Heo, K.; Lee, B.Y.; Lee, J.; Seo, D.H.; Seo, S.; Jian, J.; Hong, S. Metallic nanowire-graphene hybrid nanostructures for highly flexible field emission devices. *Nanotechnology* **2011**, *22*, 355709 DOI. 10.1088/0957-4484/22/35/355709.

[S4] Cui, Y.; Chen, J.; Zhang, X.; Lei, W.; Di, Y.; Wang, Q. Flexible field emission devices based on barium oxide nanowires. *J. Disp. Tech.* **2015**, *12*, 466–471 DOI.10.1109/JDT.2015.2500229.

[S5] Zulkifli, Z.; Kalita, G.; Tanemura, M. Fabrication of transparent and flexible carbon-doped ZnO field emission display on plastic substrate. *Phys. Status Solidi RRL* **2015**, *9*, 145–148 DOI. 10.1002/pssr.201409557.

[S6] Chen, H.; Iliev, M.N.; Liu, J.R. Ma, K.B.; Chu, W.K.; Badi, N.; Bensaoula, A.; Svedberg, E.B. Room-temperature deposition of diamond-like carbon field emitter on flexible substrates. *Nucl. Instr. Meth. Phys. Res. B* **2006**, *243*, 75–78 DOI. 10.1016/j.nimb.2005.08.119.

[S7] Cui, Y.; Chen, J.; Di, Y.; Zhang, X.; Lei, W. High performance field emission of silicon carbide nanowires and their applications in flexible field emission displays. *AIP Adv.* **2017**, *7*, 125219 DOI.10.1063/1.5012780.

[S8] Yoon, B.J.; Hong, E.H.; Jee, S.E.; Yoon, D.M.; Shim, D.S.; Son, G.Y.; Lee, Y.J.; Lee, K.H.; Kim, H.S.; Park, C.G. Fabrication of flexible carbon nanotubes field emitter arrays by direct microwave irradiation on organic polymer substrate. *J. Am. Chem. Soc.* **2005**, *127*, 8234–8235 DOI. 10.1021/ja043823n.

[S9] Ghosh, D.; Ghosh, P.; Tanemura, M.; Hayashi, A.; Hayashi, Y.; Shinji, K.; Miura, N.; Yusopa, M.Z.; Asakad, T. Highly transparent and flexible field emission devices based on single-walled carbon nanotubes films. *Chem. Commun.* **2011**, *47*, 4980 4982 DOI.10.1039/c0cc05677d.

[S10] Sankaran, K.J.; Tai, N.H.; Lin, I.N.; Flexible electron field emitters fabricated using conducting ultrananocrystalline diamond pyramidal microtips on polynorbornene films. *Appl. Phys. Lett.* **2014**, *104*, 031601 DOI: 10.1063/1.4862891.
